# Time-Staged Gamma Knife Stereotactic Radiosurgery for Large Cerebral Arteriovenous Malformations: A Preliminary Report

**DOI:** 10.1371/journal.pone.0165783

**Published:** 2016-11-02

**Authors:** Hye Ran Park, Jae Meen Lee, Jin Wook Kim, Jung-Ho Han, Hyun-Tai Chung, Moon Hee Han, Dong Gyu Kim, Sun Ha Paek

**Affiliations:** 1 Department of Neurosurgery, Soonchunhyang University Hospital, Seoul, Republic of Korea; 2 Department of Neurosurgery, Seoul National University Hospital, Seoul, Republic of Korea; 3 Department of Neurosurgery, Seoul National University Bundang Hospital, Seoul, Republic of Korea; 4 Department of Radiology, Seoul National University Hospital, Seoul, Republic of Korea; University of California Los Angeles, UNITED STATES

## Abstract

**Objective:**

We retrospectively analyzed our experience with time-staged gamma knife stereotactic radiosurgery (GKS) in treating large arteriovenous malformation(AVM)s;≥ 10 cm^3^).

**Methods:**

Forty-five patients who underwent time-staged GKS (2-stage, n = 37;3-stage,n = 8) between March 1998 and December 2011 were included. The mean volume treated was 20.42±6.29 cm^3^ (range, 10.20–38.50 cm^3^). Obliteration rates of AVMs and the associated complications after GKS were evaluated.

**Results:**

Mean AVM volume (and median marginal dose) at each GKS session in the 37 patients who underwent 2-stage GKS was 19.67±6.08 cm^3^ (13 Gy) at session 1 and 6.97±6.92 cm^3^ (17 Gy) at session 2. The median interval period was 39 months. After follow-up period of 37 months, the complete obliteration rate was 64.9%. The mean AVM volume (and median marginal dose) at each GKS session in the 8 patients who underwent 3-stage GKS was 23.90±6.50 cm^3^ (12.25 Gy), 19.43±7.46 cm^3^ (13.5 Gy), 7.48±6.86 cm^3^ (15.5 Gy) at session 1, 2, and 3, respectively. The median interval duration between each GKS session was 37.5 and 38 months, respectively. After a median follow-up period of 47.5 months, 5 patients (62.5%) achieved complete obliteration. Postradiosurgical hemorrhage developed in 5 patients (11.1%) including one case of major bleeding and 4 cases of minor bleeding. No patient suffered from clinically symptomatic radiation necrosis following radiation.

**Conclusion:**

Time-staged GKS could be an effective and safe treatment option in the management of large AVMs.

## Introduction

Arteriovenous malformation (AVM) is the second most common cause of intracerebral hemorrhage in people <35 years of age, following trauma [1]. Treatment options for cerebral AVM include surgery, endovascular treatments, and radiosurgery. Among these, stereotactic radiosurgery (SRS) has proven beneficial in the treatment of small- to medium-sized AVMs, reducing the risk of future intracranial bleeding with minimal treatment-related morbidity [2]. The cure rate in properly selected patients who undergo SRS reaches approximately 80–85%.

However, radiosurgery is generally recommended only for AVMs with an average maximum diameter ≤3 cm. The management of large AVMs remains challenging, and patients with large and, asymptomatic AVMs are usually managed conservatively because the risk associated any intervention including embolization, surgical resection, and/or radiosurgery may be equal to or greater than that arising from the natural history of the AVM if left untreated. Although SRS has been used as the alternative option, its use is limited; single-stage SRS for large volume AVMs is associated with a low rate of obliteration or unacceptable adverse radiation effects (AREs). Optimal radiation doses, considered necessary to completely obliterate the nidus with a single gamma knife stereotactic radiosurgery (GKS) session, cannot be delivered in some cases in which there is a relatively large nidus. AVMs exceeding 3 cm or 10 cm^3^ in diameter and volume, respectively are traditionally not considered appropriate candidates for radiosurgical treatment, due to their lower obliteration rates [3, 4].

In this retrospective study, we describe the outcomes of patients with large AVMs who underwent time-staged GKS at a single institution. Time-staged GKS was defined as repeated treatment of the entire nidus using low doses for each stage over an interval period of 3 to 4 years. We evaluated the obliteration rates, risk of post-GKS hemorrhage and complications.

## Patients and Methods

### Patients

This retrospective study was approved by the institutional review board of our institution (IRB No. 1508-109-696). The requirement for obtaining informed consent from the patients was waived because the study was based on the information obtained as a part of routine clinical care and medical records. We retrospectively reviewed the data of 48 patients who underwent time-staged GKS for cerebral AVMs ≥10 cm^3^ between March 1998 and December 2011. Three patients who did not undergo at least 6 months of clinical follow-up or one session of magnetic resonance imaging (MRI) were excluded. The remaining 45 patients were enrolled; their baseline characteristics at the time of initial radiosurgery are summarized in Table 1.

**Table 1:** Characteristics of the 45 patients included in this study.

Characteristics	Whole patients (n = 45)	2-staged GKS (n = 37)	3-staged GKS (n = 8)
Male/Female (n, %)	26 (57.8%) / 19 (42.2%)	21 (56.8%) / 16 (43.2%)	5 (62.5%) / 3 (37.5%)
Mean age (years)	29 ± 13 (range, 4–60)	31 ± 13 (range, 7–60)	22 ± 15 (range, 4–51)
Mean F/U duration (months)	104.9 ± 41.2 (range, 44–191)	100.1 ± 42.8 (range, 44–191)	127.0 ± 23.3 (range, 91–161)
Mean AVM volume (cm^3^)	20.42 ± 6.29 (range, 10.20–38.50)	19.67 ± 6.08 (range, 10.20–38.50)	23.90 ± 6.50 (range, 10.50–30.90)
10–14 cm^3^ (n, %)	7 (15.6%)	6 (16.2%)	1 (12.5%)
≥14 cm^3^ (n, %)	38 (84.4%)	31 (83.8%)	8 (87.5%)
Presentation			
hemorrhage (n, %)	10 (22.2%)	8 (21.6%)	2 (25%)
seizure (n, %)	9 (20.0%)	7 (18.9%)	2 (25%)
headache (n, %)	12 (26.7%)	10 (27.0%)	2 (25%)
focal transient ischemic deficits (n, %)	7 (15.6%)	6 (16.2%)	1 (12.5%)
incidental finding (n, %)	7 (15.6%)	6 (16.2%)	1 (12.5%)
Previous embolization (n, %)	6 (13.3%)	5 (13.5%)	1 (12.5%)
Previous resection (n, %)	1 (2.2%)	1 (2.7%)	0 (0%)
Location (n, %)			
hemispheric	39 (86.7%)	33 (89.2%)	6 (75%)
frontal	11 (24.4%)	9 (24.3%)	2 (25%)
parietal	11 (24.4%)	10 (27.0%)	1 (12.5%)
temporal	5 (11.1%)	5 (13.5%)	0 (0%)
occipital	2 (4.4%)	1 (2.7%)	1 (12.5%)
cerebellum	1 (2.2%)	1 (2.7%)	0 (0%)
multilobar	9 (20.0%)	7 (18.9%)	2 (25%)
deep	6 (13.3%)	4 (10.8%)	2 (25%)
brain stem	1 (2.2%)	1 (2.7%)	0 (0%)
thalamus	4 (8.9%)	2 (5.4%)	2 (25%)
basal ganglia	1 (2.2%)	1 (2.7%)	0 (0%)
Coexisting aneurysm (n, %)	7 (15.6%)	7 (18.9%)	0 (0%)
perinidal	2 (4.4%)	2 (5.4%)	
intranidal	0 (0%)	0 (0%)	
unrelated	5 (11.1%)	5 (13.5%)	
AVM nidus			
Diffuse	29 (64.4%)	25 (67.6%)	4 (50%)
Compact	16 (35.6%)	12 (32.4%)	4 (50%)
Spetzler-Martin Grade (n, %)			
1–2	15 (33.3%)	12 (32.4%)	3 (37.5%)
3	17 (37.8%)	16 (43.2%)	1 (12.5%)
4	11 (24.4%)	8 (21.6%)	3 (37.5%)
5	2 (4.4%)	1 (2.7%)	1 (12.5%)
RBAS *			
Median (range)	2.60 (1.37–4.77)	2.52 (1.70–4.77)	3.06 (1.37–3.47)
1.01–2.00 (n, %)	7 (15.6%)	7 (18.9%)	0
2.01–3.00 (n, %)	25 (55.6%)	21 (56.8%)	4 (50%)
3.01–4.00 (n, %)	12 (26.7%)	8 (21.6%)	4 (50%)
4.01–5.00 (n, %)	1 (2.2%)	1 (2.7%)	0

*Radiorusrgery-based AVM scale = (0.1)(volume,mL) + (0.02)(age,yr)+(0.3)(location, hemispheric/corpus callosum/cerebellar = 0; basal ganglia/thalamus/brainstem = 1)[9]

The patients were carefully selected for radiosurgery by experts in neurosurgery and radiosurgery. Radiosurgery was recommended for those considered to have high rates of morbidity or mortality associated with surgical resection under general anesthesia.

### Radiosurgical technique

Radiosurgery was performed in patients under local anesthesia, supplemented with intravenous sedation. Patients were treated using the Leksell Gamma Knife (model B or C, Elekta Instrument AB) with Leksell Gamma Plan (Elekta, Stockholm, Sweden). Treatment planning was based on the combination of stereotactic biplanar angiography and thin-slice MRI. Image-integrated treatment planning was performed by neurosurgeons and interventional neuroradiologists.

The GKS isodose, maximum dose, and marginal dose were initially decided based on AVM volume and were calculated during dose planning with the best-fit isodose method. When selecting a radiation dose, we referred to the guideline of the Kjellberg 1% isoeffective line for cerebral necrosis after proton-beam radiosurgery [5], and optimized the dose by reducing it based on the location of the AVM. The prescribed dose was decreased preferably to nearly 10 Gy for large (volumes ≥10 cm^3^) and extra-large (volumes ≥14 cm^3^) AVMs, which was used as the lowest dose to obtain any obliteration response using GKS [6]. The treatments were designed to deliver 50% of the maximum dose to the margins of the lesion.

### Follow-up evaluations

Patients were clinically evaluated at 6 months, 1, 2, and 3 years after GKS. MRI was recommended every 6 months. Angiography was recommended at 3 years after radiosurgery, but it was performed earlier in patients whose AVM nidus had disappeared on MRI. Complete AVM obliteration was defined as an absence of abnormal vessels in the former nidus of the AVM, the disappearance or normalization of draining veins, and normal circulation time based on the result of angiography. In patients who did not undergo angiography, AVM obliteration was defined as a disappearance of the nidus in enhanced T1- and T2-weighted images and an absence of flow-void signal abnormalities in T2-weighted images on MRI. We determined AVM nidus obliteration based on both MRI and angiography. If the neuroimaging study performed 3 years after GKS revealed a residual nidus, repeated radiosurgical treatment was simultaneously recommended with consideration for the size and location of the residual AVM. The follow-up protocol and definition of outcome used for repeated treatments were the same as those for primary AVM radiosurgery. The final follow-up imaging modality since the last GKS session was MRI in 22 patients (48.9%) and angiographyin 23 patients (51.1%).

Post-GKS hemorrhage was defined as a clinically symptomatic event such as headache, seizure, or loss of consciousness, or focal deficits along with signs of acute hemorrhage detected by means of computed tomography (CT) or MRI. PRI changes included postradiosurgical edema, radiation necrosis, and delayed cyst formation on T2-weighted and/or FLAIR MRI. Information about all patients was recorded in a radiosurgical database at the time of their treatment and at each clinical follow-up visit.

### Statistical analysis

Statistical analyses were performed using SPSS version 18.0 (SPSS, Inc., Chicago, IL, USA). A two-sided *P* value of < 0.05 was considered to indicate statistical significance.

## Results

### Two-stage GKS

**Fig 1:**
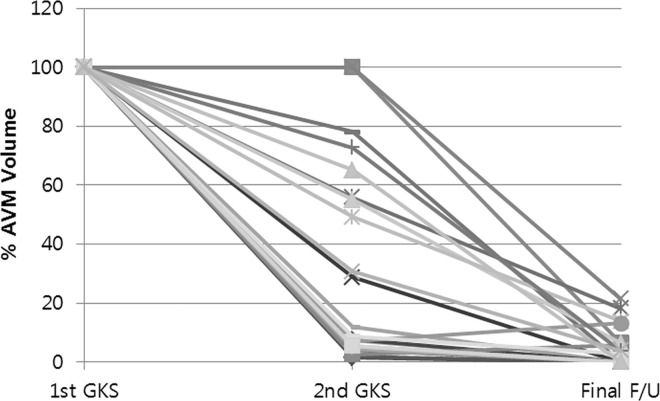
The volume percent at each gamma knife radiosurgery (GKS) session of the 37 patients who underwent 2-stage GKS

Table 2 summarizes the radiosurgery parameters at each GKS sessions. Thirty-seven patients underwent 2-stage GKS for a large AVM. Before radiosurgery, the mean AVM volume in the 37 patients was 19.67 ± 6.08 cm^3^ (range, 10.20–38.50 cm^3^). The median radiosurgery parameters at the 1^st^ GKS session were: marginal dose 13 Gy (range, 8–16), isodose line 50%, shot 14 (range, 8–30), and covered ratio 94% (range, 88–98). After median interval duration of 39 months (range, 31–113) between the 1^st^ and 2^nd^ GKS sessions, the mean volume of the remnant AVM was 6.97 ± 6.92 cm^3^ (range, 0.37–25.1 cm^3^). The mean remnant nidus volume percent compared to the initial volume was 43.63 ± 37.28%. The patients underwent the 2^nd^ GKS session with the following median parameters: marginal dose 17 Gy (range, 7–21), isodose line 50%, shot 12 (range, 3–31), and covered ratio 97% (range, 93–99). The median interval between the 2^nd^ GKS session and the final imaging follow-up was 44 months (range, 6–136). Among these 37 patients, 24 patients (64.9%) obtained complete obliteration. The remnant nidus volume percent compared to the initial volume in the remaining 13 patients was 10.9 ± 6.5%. These 13 patients who failed to achieve complete obliteration obtained considerable volume reduction. Fig 1 presents the volume percent at each GKS session for individual patients.

**Table 2:** GKS parameters at each GKS sessions.

	2-stage GKS (n = 37)		3-stage GKS (n = 8)		
	1^st^ session	2^nd^ session	1^st^ session	2^nd^ session	3^rd^ session
Treatment volume, mean (cm^3^)	19.67 ± 6.08 (range, 10.20–38.50)	6.97 ± 6.92(range, 0.37–25.1)	23.90 ± 6.50 (range, 10.50–30.90)	19.43 ± 7.46 (range, 10.50–28.20)	7.48 ± 6.86 (range, 0.54–17.41)
Marginal dose, median (Gy)	13.0 (range, 8–16)	17.0 (range, 7–21)	12.25 (range, 10–15)	13.5 (range, 12–15)	15.5 (range, 12.5–18.0)
Isodose line, median (%)	50 (range, 50)	50 (range, 50)	50 (range, 49–50)	50 (range, 49–50)	50 (range, 50)
Shot, median	14 (range, 8–30)	12 (range, 3–31)	17 (range, 11–34)	17 (range, 8–34)	18.5 (range, 7–23)
Covered ratio, median (%)	94.0 (range, 88.0–98.0)	97.0 (range, 93.0–99.0)	94.0 (range, 59.0–96.0)	94.0 (range, 90.0–96.0)	96.5 (range, 95.0–99.0)

### Three-stage GKS

**Fig 2:**
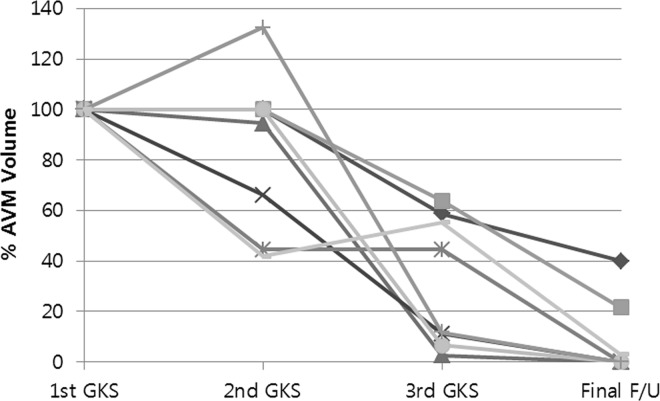
The volume percent at each gamma knife radiosurgery (GKS) session of the 8 patients who underwent 3-stage GKS

Five patients underwent 3-stage GKS for AVM with a volume ≥10 cm^3^. The mean AVM volume at the time of each GKS session was as follows: 1^st^ GKS session, 23.90 ± 6.50 cm^3^ (range, 10.50–7.46 cm^3^); 2^nd^ GKS session, 19.43 ± 7.46 cm^3^ (range, 10.50–28.20 cm^3^), 3^rd^ GKS session, 7.48 ± 6.86 cm^3^ (range, 0.54–17.41 cm^3^). The median interval durations between the 1^st^ and 2^nd^ GKS sessions, and the 2^nd^ and 3^rd^ GKS sessions were 37.5 (range, 32–46) and 38 (range, 35–68) months, respectively. The marginal dose in each GKS sessions gradually increased; 1^st^ GKS session, 12.25 Gy (range, 10–15); 2^nd^ GKS session, 13.5 Gy (range, 12–15); 3^rd^ GKS session, 15.5 Gy (range 12.5–18). Finally, after a median follow-up duration of 47.5 months (range, 15–70) following the 3^rd^ GKS session, 5 patients achieved complete obliteration (complete obliteration rate, 62.5%). The remaining 3 patients also experienced substantial volume reduction; the remnant nidus volume percent compared to the initial volume was 39.8%, 21.5%, and 2.98%, respectively. Fig 2 presents the volume percent at each GKS session for these five patients. One patient experienced transient volume increase after the 1^st^ GKS session without any relevant symptom. This transient volume increase was thought to be caused by the change of flow dynamics within AVM, and the following imaging study revealed decrease of AVM nidus.

### Overall treatment response and clinical outcome

The overall obliteration rate of AVM nidus was 64.4%, and the remnant nidus volume percent compared to the initial volume was 12.9 ± 9.9% (range, 2.8–39.8) in the non-obliteration group. The median period between the 1^st^ GKS session and AVM obliteration was 76.50 ± 28.03 months (range, 39–173) in the 2-stage GKS group and 115.0 ± 13.8 months (range, 90–128) in the 3-stage GKS group. Coexisting aneurysm was the only significant factor correlated with AVM obliteration (P = 0.013, Exp(B) = 0.038, multivariate analysis) in both univariate and multivariate analysis (Table A in S1 Table).

The seizure-free and seizure medication-free rate was 84.4% and 68.9%, respectively. Table B in S1 Table summarizes the prognostic factors associated with seizure-free and seizure-free outcome. There was no factor that significantly correlated with seizure freedom. However, AVM nidus obliteration was significantly associated with being seizure medication-free (P = 0.023, Exp(B) = 0.023, multivariate analysis) in both univariate and multivariate analysis. Of the 9 patients who presented with seizure as the initial presentation, 6 (66.7%) obtained seizure-free status of Engel classification I. The remaining 3 patients are taking anti-epilepsy drugs.

The mean final KPS score was 95.33 ± 15.46 points, and 40 patients (88.9%) returned to their work at the final clinical follow-up points. Table 3 represents pre- and post-treatment mRS (modified Rankin Scale) scores. Twenty-nine patients (64.4%) showed no change on mRS. Eleven patients (24.4%) had higher mRS scores, and 5 patients (11.1%) had improvement after treatment.

**Table 3:** Pre- and Post-treatment modified Rankin Scores.

Pre mRS score	Post mRS score						
	0	1	2	3	4	5	6
0	25	4	0	0	0	0	0
1	9	4	0	0	0	0	1
2	1	1	0	0	0	0	0
3	0	0	0	0	0	0	0
4	0	0	0	0	0	0	0
5	0	0	0	0	0	0	0
6	0	0	0	0	0	0	0

We referred Pollock and Flickinger’s outcome criteria and stratified the outcomes. [7, 8] Patients’ outcome was stratified as excellent, good, fair, unchanged, poor, or death, based on the last follow-up review. Twenty-one patients (47%) had an excellent outcome which defined as complete nidus obliteration and no new development of neurological deficit. Six patients (13%) and 2 patients (4%) with complete nidus obliteration were classified as a good outcome with minor deficit that did not interfere with normal daily life and a fair outcome with major deficit causing a decline in the patient’s level of functioning, respectively. Fifteen patients (33%) had an unchanged outcome which was defined as persistent arteriovenous shunting without new neurological deficit. No patient was classified as poor outcome with a new neurological deficit and incomplete nidus obliteration. One patient (2%) died.

**Fig 3:**
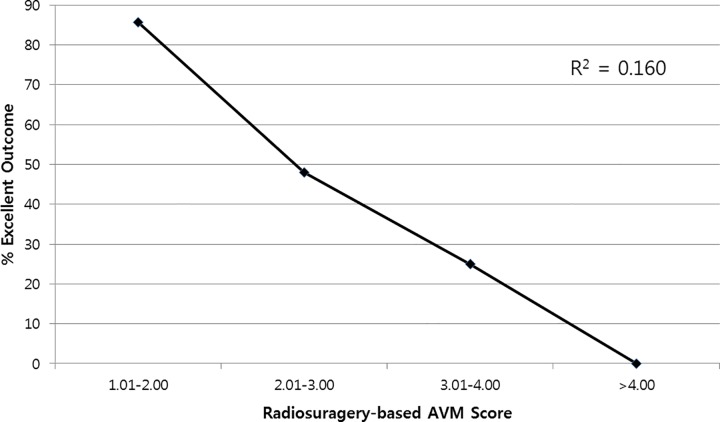
Graph showing relationship between modified radiosurgery-based AVM score (RBAS) and the percentage of patients who obtained an excellent outcome at the last follow-up. R2 = 0.160, p = 0.006.

The median modified radiosurgery-based AVM score (RBAS) was 2.60 (range, 1.37–4.77).[4, 8, 9] Univariate linear regression showed a statistically significant correlation between RBAS and excellent outcome (1.01–2.00, 85.7%; 2.01–3.00, 48.0%; 3.01–4.00, 25.0%; >4.00, 0%; F = 11.200, R^2^ = 0.160, *P* = 0.006; Fig 3).

### Post-GKS complications and additional treatment after GKS

Complications included post-GKS hemorrhage (5 patients, 11.1%) and post-radiosurgical imaging change (19 patients, 42.2%). The median interval period between the 1^st^ GKS session and the occurrence of hemorrhage was 54 ± 43 months (range, 32–140). Except for one patient who died of intracerebral hemorrhage, the other four patients experienced minor bleeding and recovered after management. One patient harbored intraventricular and intracerebral hemorrhage 27 months after the 2^nd^ GKS session, and emergent extraventricular drainage (EVD) was performed. However, angiography performed 17 days after hemorrhage occurrence revealed no remnant AVM nidus. Another patient received ventriculo-peritoneal shunt due to combined hydrocephalus lead performed normal daily life. Stereotactic hematoma removal was required in one patient, and he achieved full recovery. Post-radiosurgical imaging changes included postradiosurgical edema, radiation necrosis, and delayed cyst formation on T2-weighted and/or FLAIR MR Images. Any clinically meaningful event associated with post-radiosurgical imaging change was not noted.

## Discussion

### Management of large AVM

A Randomized Trial of Unruptured Brain AVMs (ARUBA) was a prospective, multicenter trial that randomized 223 patients with unruptured AVMs to medical or interventional therapy. ARUBA was terminated early because interventional therapy showed an inferior outcome compared with medical treatment [10]. However, considering the higher risk of future hemorrhage and associated morbidity of a large AVM, if an alternative optimal treatment option can be developed, the benefit of treating large AVMs might outweigh the risk of treatment.

Surgical resection is the widely used treatment option for AVMs. It provides a distinct advantage of complete removal in a single or staged operation. However, surgery is far less attractive for large AVMs due to higher rates of morbidity and mortality. Additionally, there is an inherent risk of normal perfusion pressure breakthrough with larger lesions, and the risk of future hemorrhage is increased in partially treated lesions [11]. For this reason, surgery is not solely performed for treating large AVMs without other supportive managements.

The reported rates of complete radiological obliteration of AVMs in patients treated with SRS ranges from 50% to 80% [4, 12]. However, the outcomes of SRS for large AVMs have been disappointing. The patients with a larger AVM tended to show lower obliteration rates than those with a smaller AVM [13]. Single-session radiosurgery often does not yield a complete cure, and so is not recommended for lesions ≥3 cm in diameter or ≥10 cm^3^ in volume by most centers [3]. Higher complication rates for large AVMs after GKS, such as post-radiosurgical MRI abnormalities and radiation necrosis have been reported, because of the higher dose needed to cover the lesion [14, 15].

Previous authors have reported postradiosurgical complications including symptomatic sequelae (9%-11%), postirradiation imaging change up to 30% such as white matter disease, edema, or necrosis, and post-radiosurgical hemorrhage [4]. The risk of radiation injury resulting in a permanent neurologic deficit is reportedly 2% to 3% for single-session SRS for AVMs [16]. Han et al. demonstrated an annual risk of 10.4% for patients who received incomplete treatment [17]. One of the major drawbacks of SRS for AVMs is the presence of a latency period (mean, 2–3 years) before full biologic effects occur. During this latency period, partial treatment results in compartmental cytoarchitectural changes, and the risk of hemorrhage still persists. The risk of secondary malignancy after SRS is less than 1/1000 with a follow-up duration of more than 5 years [18].

To overcome the forementioned limitations of single-session SRS for AVM, a new approach including staged GKS has been attempted. Volume-staged radiosurgery has been regarded as an alternative option for single modality treatment [19]. Pollock et al. targeted different volume compartments, and performed staged radiosurgery for small, medium, and large AVMs. They compared volume-staged SRS to hypothetical single-session procedures and found that volume staging resulted in less radiation exposure to the adjacent brain [20].

The disadvantage of volume-staged GKS is a longer latency period between treatments [21]. Volume-staged GKS is considered to be associated with greater risk of hemorrhage due to the latency period of 3–6 months. An increased risk of hemorrhage resulting from redistribution of blood flow to non-irradiated regions has been described [22].

### Time-staged GKS for treating a large AVM

**Fig 4:**
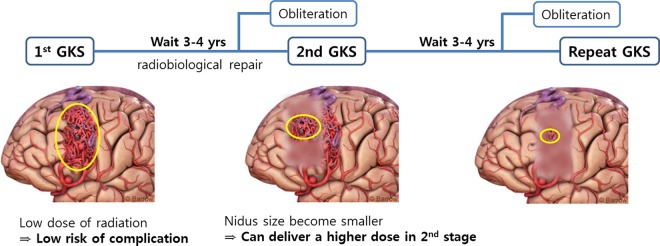
The schematic illustration of time-staged gamma knife radiosurgery (GKS) for large arteriovenous malformation (AVM)

The dilemma in radiosurgical treatment of large AVM is the inverse proportion relationship between the obliteration and the complication according to the prescribed dose. Time-staged GKS was introduced to achieve dose reduction in order to reduce the adverse radiation effects. After the 1^st^ GKS session with a low dose of 12–13 Gy covering the whole lesion, there is a waiting period of about 3–4 years. The occurrence of radiobiological repair is expected during this waiting period. Theoretically, post-irradiation radiobiological repair may occur quite quickly; hence, radiosurgical treatment can be repeated within only 3–6 months [19, 23]. The nidus is expected to become smaller during this waiting period. Then, a higher dose can be safely allowed for the reduced lesion in the next GKS session. Fig 4 presents this concept of time-staged GKS for treating a large AVM. Yamamoto et al. used this strategy with an interval of at least 3 years between procedures to avoid complicated dose-planning procedures[24]. They reported that complete obliteration occurred 3 or more years after irradiation in some patients, even on using a low dose.

The main advantage of time-staged GKS is that a suboptimal dose <1% complication line of Kjellberg et al. can be used [25]. The suboptimal dose radiosurgery strategy induces less hemodynamic stress by blood flow redistribution, compared with volume-staged radiosurgery [22]. This might be the reason for the low complication rate in this study. The problem with time-staged GKS is that the risk of hemorrhage still persists during the waiting period. Yamamoto et al. reported an incidence of hemorrhage of 22.6% [24]. Although there have been many reports about the decreased hemorrhage risk during the latency period after GKS [26, 27], an unchanged or even increased hemorrhage risk compared with the natural course of the disease has been reported [28]. If an adequate dose of radiation is delivered to the entire lesion, the risk of hemorrhage might be reduced before obliteration [25, 26].

The distinction between the concept of repeated GKS and time-staged GKS might be a moot point. In our opinion, these procedural concepts could be distinguished by the intention at the time of the 1^st^ GKS session. The patients included in this study underwent time-staged GKS, intentionally. On the other hand, repeated GKS is aimed at residual AVMs which failed to achieve complete obliteration at the prior GKS session. There have been a few reports about repeated GKS [29, 30]. The authors concluded that repeated GKS was relatively safe and effective, and the prescribed dose should not be reduced for repeated radiosurgery compared to initial treatment.

### Seizure control

Radiosurgery including GKS has shown favorable seizure outcomes for patients with AVM. In a literature review, the mean seizure-free rate following radiosurgery was 53.4% (ranges, 0%-95%) [31]. The effect of radiosurgery on seizure is known to occur directly by the restriction of epileptic activity though irradiation of gliotic capsule around the nidus, and indirectly by reduction of blood flow by the arteriovenous shunts resulting in the ischemia in the surrounding tissue [32].

Consistent with the previously published articles, the authors experienced a favorable seizure outcome. These favorable results in terms of seizure control lead to a satisfactory performance status and return to daily life activities. Conclusively, time-staged GKS was effective for seizure control, and may improve the quality of life and employment status in the patients with a large AVM.

### Limitations

This study has limitations as a preliminary interim report a retrospective study design. The effect of GKS should be assessed in a large randomized cohort, with comparison to a group receiving no treatment. The small cohort size, retrospective study design, and lack of long-term follow-up after the final GKS session limit the power of this study.

## Conclusion

Proper management is required for large AVMs. Time-staged GKS might be an effective and safe treatment option in the management of large AVMs. Assessment of the long-term outcome in a larger cohort is required for the validation of time-staged GKS in cases of large unruptured AVM.

## Supporting Information

S1 Table Table A. Prognostic factors associated with AVM obliteration, Post-GKS hemorrhage, and PRI change. Table B. Prognostic factors associated with seizure-free and seizure medication-free.
(DOCX)
