# Supplementary material for: Time-Staged Gamma Knife Stereotactic Radiosurgery for Large Cerebral Arteriovenous Malformations: A Preliminary Report
Source: PLoS One. 2016 Nov 2;11(11):e0165783. doi: 10.1371/journal.pone.0165783 (PMC5091869; doi:10.1371/journal.pone.0165783)
Supplement: S1 Table — Table A. Prognostic factors associated with AVM obliteration, Post-GKS hemorrhage, and PRI change. Table B. Prognostic factors associated with seizure-free and seizure medication-free. (DOCX) [file pone.0165783.s001.docx]

Table A. Prognostic factors associated with AVM obliteration, Post-GKS hemorrhage, and PRI change

| Variable | AVM obliteration | | Post-GKS hemorrhage | | PRI change | |
| --- | --- | --- | --- | --- | --- | --- |
|  | Univariate | Multivariate | Univariate | Multivariate | Univariate | Multivariate |
| Age ≥ 17 years | 0.496 | 0.837 | 0.999 | 0.999 | 0.624 | 0.544 |
| Sex : Female | 0.161 | 0.265 | 0.308 | 0.391 | 0.989 | 0.834 |
| AVM nidus : deep location | 0.319 | 0.715 | 0.645 | 0.326 | 0.680 | 0.224 |
| Coexisting aneurysm | **0.013 (Exp(B) 0.060) 95% CI 0.006-0.557** | **0.013 (Exp(B) 0.038) 95% CI 0.003-0.494** | 0.999 | 0.999 | 0.970 | 0.452 |
| AVM volume ≥ 14 cm^3^ | 0.676 | 0.907 | 0.772 | 0.941 | 0.390 | 0.265 |
| Previous embolization | 0.903 | 0.907 | 0.645 | 0.526 | 0.638 | 0.785 |
| Initial hemorrhagic manifestation | 0.256 | 0.152 | 0.324 | 0.069 | 0.122 | 0.122 |
| Previous resection | 1.000 | 1.000 | 1.000 | 1.000 | 1.000 | 1.000 |
| PRI change | 0.878 | 0.850 | 0.402 | 0.242 |  |  |

Table B. Prognostic factors associated with seizure-free and seizure medication-free

| Variable | Seizure free | | Seizure-medication free | |
| --- | --- | --- | --- | --- |
|  | Univariate | Multivariate | Univariate | Multivariate |
| Age ≥ 17 years | 0.076 | 0.160 | 0.213 | 0.182 |
| Sex : Female | 0.970 | 0.998 | 0.554 | 0.711 |
| AVM nidus deep location | 0.999 | 0.999 | 0.424 | 0.153 |
| Initial seizure presentation | 0.117 | 0.997 | 0.340 | 0.706 |
| AVM volume ≥ 14 cm^3^ | 0.920 | 0.645 | 0.469 | 0.475 |
| Previous embolization | 0.936 | 1.000 | 0.900 | 0.565 |
| Previous resection | 1.000 | 1.000 | 1.000 | 1.000 |
| AVM nidus obliteration | 0.998 | 0.997 | **0.023 (Exp(B) 0.082) 95% CI 0.010-0.706** | **0.023 (Exp(B) 0.023) 95% CI 0.010-0.706** |
| Post-GKS hemorrhage | 0.135 | 0.997 | 0.160 | 0.214 |
| PRI change | 0.970 | 0.273 | 0.178 | 0.324 |
